# Osteogenesis of Human iPSC-Derived MSCs by PLLA/SF Nanofiber Scaffolds Loaded with Extracellular Matrix

**DOI:** 10.1155/2023/5280613

**Published:** 2023-02-06

**Authors:** Junming Zhang, Lingbin Che, Yunliang Wu, Lei Zhou, Li Liu, Yuanhang Yue, Dianwen Song, Xiangxin Lou

**Affiliations:** ^1^College of Biological Science and Medical Engineering, Donghua University, Shanghai, China; ^2^Department of Orthopedics, Shanghai General Hospital, School of Medicine, Shanghai Jiaotong University, Shanghai, China

## Abstract

Bone defects that arise from trauma, skeletal diseases, or tumor resections have become the commonest and most thorny problems in orthopedic clinics. Recently, biocomposite materials used as artificial bone repair materials have provided a promising approach for bone regeneration. In this study, poly (l-lactide acid) (PLLA) and silk fibroin (SF) were used to fabricate nanofiber scaffolds by electrospinning technology. In order to simulate a biomimetic osteoblast microenvironment, decellularized extracellular matrix from osteoblasts was loaded into the biocomposite scaffolds (O-ECM/PLLA/SF). It was found that the O-ECM/PLLA/SF scaffolds were nontoxic for L929 cells and had good cytocompatibility. Their effects on mesenchymal stem cells derived from human-induced pluripotent stem cell (iPSC-MSC) behavior were investigated. As a result, the scaffolds with the addition of O-ECM showed enhanced alizarin red S (ARS) activity. In addition, higher expression of osteogenic gene markers such as runt-related transcription factor 2 (Runx2), collagen type I (Col-1), and osteocalcin (OCN) as well as upregulated expression of osteogenic marker protein osteopontin (OPN) and Col-1 further substantiated the applicability of O-ECM/PLLA/SF scaffolds for osteogenesis. Furthermore, the *in vivo* study also indicated maximal new bone formation in the skull defect model of Sprague Dawley (SD) rats treated with the O-ECM/PLLA/SF carried by human iPSC-MSCs. Hence, this study suggests that O-ECM/PLLA/SF scaffolds have a potential application in bone tissue engineering.

## 1. Introduction

Although bone tissue has a natural regenerative power in small site of damage, the bone defect site cannot be repaired and reconstructed when the defect area exceeds a certain range [1, 2]. Currently, autogenous bone transplantation is an effective treatment for critical-sized bone defects [3]. Nevertheless, the autologous bone grafting has inevitable disadvantages such as limited bone sources [4] and a high complication rate [5]. Recently, bone tissue engineering (BTE) approaches have held promise in clinical therapy.

The promising BTE approach for bone repair is to mimic the bone growth environment by creating a biomimetic microenvironment for cell adhesion, proliferation, and osteogenic differentiation [6]. Electrospinning is a convenient and versatile technique to create 2D or 3D nanofiber scaffolds that have similar dimensions compared to extracellular matrix (ECM). Poly (l-lactide acid) (PLLA) is a biobased crystalline polymer originated from renewable resources with a nontoxic degradation component, which is widely used in the field of tissue engineering technology [7, 8]. But pure PLLA scaffold, which has some disadvantages including poor bio-mechanical property and high hydrophobicity, is not an ideal biological material [9]. Silk fibroin (SF) has been widely used for biomedical applications due to its outstanding mechanical properties and good oxygen and water vapour permeability [10, 11]. Recently, some studies showed that the PLLA/SF composite nanofiber scaffold promotes progenitor stem cells differentiation into osteoblasts, proliferation, mineralization, and ossification process [12, 13].

Ideal BTE scaffolds should mimic the architecture of the native bony ECM [14]. However, traditional electrospinning can only produce membrane biomaterials lacking natural ECM that are essential for cell behavior and tissue regeneration [15]. The interactions between ECM and cells have a great influence on wound repair by providing provisional matrix proteins at the early stage of wound healing [16]. The ECM is decellularized and processed into different forms to take advantage of its inherent structural and biochemical properties, which are essential for tissue regeneration [17]. In theory, acellular matrix from osteoblasts is more conducive to promoting cell osteogenic differentiation and bone regeneration.

As an alternative stem cell source, recently, a new type of mesenchymal stem cells (MSCs) which were derived from induced pluripotent stem cells (iPSC-MSCs) that morphologically and functionally resemble MSCs, have the potential to differentiate into osteoblasts under appropriate conditions [18]. Therefore, iPSC-MSCs can be considered as a promising source of seed cells for BTE. However, how to control the differentiation of iPSC-MSCs into functional osteoblasts *in vitro* and *in vivo* is still poorly understood.

**Figure 1:**
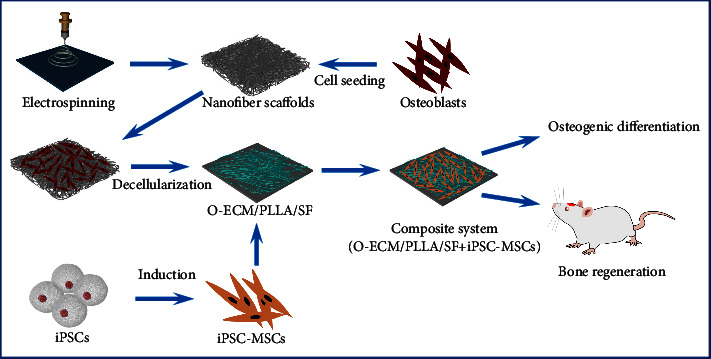
Schematic diagrams of preparation of the “O-ECM/PLLA/SF + iPSC-MSCs” composite system for osteogenic differentiation *in vitro* and *in vivo*. O-ECM, ECM derived from osteoblasts; iPSCs, induced pluripotent stem cells; iPSC-MSCs, iPSC-derived mesenchymal stem cells.

In this study, electrospun nanofibrous scaffolds loaded with decellularized ECM from osteoblasts (O-ECM/PLLA/SF) were fabricated to explore their potential application in bone tissue engineering. First, prefabricated electrospun PLLA/SF nanofiber scaffolds were processed and then coated with O-ECM to obtain O-ECM/PLLA/SF nanofibrous scaffolds. Second, MSCs were obtained from human induced pluripotent stem cells. Finally, the effect of O-ECM/PLLA/SF functionalization on osteogenic differentiation of iPSC-MSCs was evaluated. We investigated the micromorphology, cell viability, and osteogenic differentiation of iPSC-MSCs *in vitro*. Furthermore, the ability of stem cells and scaffold complex to promote bone regeneration *in vivo* was evaluated in a critical-sized rat cranial bone defect model (Figure 1). We hypothesized that the existence of the natural O-ECM could collaboratively promote osteoinduction by the biomaterial scaffolds.

## 2. Materials and Methods

### 2.1. Materials

PLLA (Mw = 100,000 Da) was obtained from Daigang Biomaterial Co., Ltd. (Jinan, China). Silkworm cocoon was purchased from Hebei Baoding Chinese Medicinal Materials Company (Hebei, China). Fetal bovine serum (FBS), trypsin-ethylenediaminetetraacetic acid (EDTA), penicillin/streptomycin, L-glutamine, and Dulbecco's Modified Eagle Medium/Nutrient Mixture F-12 (DMEM/F12) were purchased from Gibco (Gibco, Carlsbad, CA). Cell Counting Kit-8 and paraformaldehyde (PFA) were supplied by MesGen Biotech (Shanghai, China). Triton X-100 and NH_4_OH were purchased from Sinopharm Chemical Reagent Co., Ltd. (Shanghai, China).

Sprague–Dawley rats and BALB/C nude mice were provided by the Shanghai Slac Laboratory Animal Co., Ltd. (Shanghai, China). L929 cells and iPSCs (catalogue number: SCSP-1301) were supplied by the Chinese Academy of Sciences (Shanghai, China). The Animal Research Committee of Donghua University approved all experimental protocols, which were performed in accordance with the National Institutes of Health Guidelines for the Care and Use of Laboratory Animals.

### 2.2. Electrospinning of PLLA/SF Nanofiber Scaffold

The extraction of SF mainly followed the procedure described previously [19]. In brief, silk cocoons were washed, dried at 40°C for 8 h, and degummed in 1 wt% Na_2_CO_3_. The extracted SF was dissolved in CaCl_2_ : CH_3_CH_2_OH : H_2_O solution (mole ratio is 1 : 2 : 8) at 72°C for 1 h. SF solution was obtained after dialyzing of 3–6 days followed by filtration. PLLA/SF nanofibrous scaffolds were prepared by the electrospinning technique following the procedure described previously [20]. PLLA/SF with the different mass ratios including 100/0, 70/30, 50/50, 300/70, and 0/100 were dissolved in HFIP solvent to prepare polymer solutions with a fixed concentration of 8% (w/v), respectively. The polymer solutions were further loaded into a 10 mL syringe. The solution flow rates were maintained at 1 mL/h. A high voltage of 12 kV was applied to generate the polymer jet. The samples were then stored at 4°C for further study.

### 2.3. Characterization of Scaffolds

Characterization of different ratios of PLLA/SF composite scaffolds was conducted to select the most appropriate scaffold.

#### 2.3.1. Scanning Electron Microscopy (SEM)

The microstructure of PLLA/SF scaffolds with the different mass ratios was observed using a scanning electron microscope (JSM-5600, JEOL, Japan) with a voltage of 10–15 kV. The various nanofibrous scaffolds (*n* = 3) were cut into 1 × 1 mm pieces and stuck on the platform with conductive adhesive, then sputter-coated with gold for 60 s before scanning with SEM. The diameter of fibers (*n* = 100) was analyzed by the Image J software (National Institutes of Health, USA). Statistical analysis was processed using the Origin 8.0 statistical software (OriginLab, USA).

#### 2.3.2. Fourier Transform Infrared (FTIR) Spectroscopy

The PLLA/SF scaffolds with the different mass ratios (*n* = 3) were cut into 1 × 1 mm pieces. The chemical structure of various nanofibrous scaffolds was analyzed by FTIR. FTIR spectra were obtained with a Nicolet NEXUS-670. The FTIR spectra ranged from 4000 cm^−1^ to 400 cm^−1^, with 4 cm^−1^ wavenumber resolution and an average measurement of 32 scans. The statistical analysis was processed using the Origin 8.0 statistical software (OriginLab).

#### 2.3.3. Cytocompatibility Analysis

The various nanofibrous scaffolds were manufactured into disk samples with a diameter of 15 mm and a weight of 4 mg that fit the wells of a 24-well cell culture plate with scissors. Then, the scaffolds were sterilized under ultraviolet light for 2 h and washed with phosphate buffered saline (PBS) 3 times. L929 cells at a density of 2 × 10^4^ cells/mL were seeded into the scaffolds with a culture medium (DMEM/F12 + 10% FBS + 1% penicillin/streptomycin) at 37°C under 5% CO_2_ atmosphere. As a control, the same L929 cells were seeded on tissue culture plates (TCP) with a culture medium (DMEM/F12 + 10% FBS + 1% penicillin/streptomycin) at 37°C under 5% CO_2_ atmosphere. After culturing for 1, 4, and 7 days, Cell Counting Kit-8 working solution was added into the scaffolds. After incubation for 2 h at 37°C, the optical density (OD) values were recorded at 450 nm by a plate reader (Multiskan MK3, Thermo, USA), which can indirectly reflect the number of living cells.

### 2.4. Extraction and Culture of Osteoblasts and Building O-ECM/PLLA/SF Composite Scaffolds

The osteoblasts were isolated by the method described previously [21]. In brief, calvarial fragments from neonatal SD rats were collected shortly after delivery. The periosteum was removed, and bone fragments were placed on the tissue culture plates and digested with 0.25% trypsin-EDTA in an incubator at 37°C under 5% CO_2_ atmosphere for 30 min. The collected osteoblasts were cultured in DMEM/F12 supplemented with 10% FBS, 2 mM L-glutamine, and 1% penicillin/streptomycin in a humidified atmosphere containing 5% CO_2_ at 37°C. PLLA/SF scaffolds were coated with O-ECM as described previously [22]. In brief, the osteoblasts at passage 3 (P3) were cultured on the PLLA/SF nanofiber scaffolds placed in DMEM/F12 supplemented with 10% FBS, 2 mM L-glutamine, and 1% penicillin/streptomycin for 6 days. The culture medium was replaced with DMEM/F12 supplemented with 10% FBS, 2 mM L-glutamine, 1% penicillin/streptomycin, 50 *μ*M ascorbic acid (Sigma, USA), and the osteoblasts were cultured for another 4 days. Next, the culture medium was removed, and cells were treated with a solution containing 0.5% Triton X-100 and 20 mM NH_4_OH at room temperature for 5 min. Finally, the samples were rinsed with PBS 3 times and then stored at 4°C for further study.

### 2.5. Generation and Evaluation of iPSC-MSCs

Neonatal foreskin cells were induced into iPSCs, and the iPSCs were induced by reprogramming transcription factors as OCT4, SOX2, KLF4, and MYC. The feeder-free system was used during the culture of iPSCs. The human iPSCs were induced to differentiate into MSCs by the method described previously with minor modifications [23]. Briefly, iPSCs at passage 14 (P14) were collected by 0.25% trypsin-EDTA and transferred to low-attachment cell culture dishes in the mTeSR^TM^1 culture medium (Stem Cell, Canada) to form embryoid bodies (EBs). After 2 days, floating EBs were collected by centrifugation and cultured in mTeSR^TM^1 culture medium supplemented with 1 *μ*M all trans retinoic acid (RA, Sigma) for another 2 days. Then, the EBs were collected and plated onto 0.1% gelatin-coated 6-well cell culture plates in DMEM/F12 culture medium supplemented with 10% FBS and 2 mM L-glutamine. The new cells were collected and cultured for up to 7 days. These differentiated cells were designated as iPSC-MSCs.

#### 2.5.1. Flow Cytometry

iPSC-MSCs were collected by 0.25% trypsin-EDTA, and single cell suspensions of P7 at a density of 1 × 10^6^ cells/mL were incubated with phycoerythrin (PE)-conjugated mouse anti-human monoclonal antibodies CD29, CD44, and CD90 (1 : 500, all from BD Biosciences, USA) and fluorescein isothiocyanate (FITC)-conjugated mouse anti-human monoclonal antibodies CD34 and CD45 (1 : 500, all from BD Biosciences, USA) for 30 min at 4°C. These cells were resuspended with 500 *μ*L PBS and analyzed using the FACS Calibur (Becton Dickinson, USA). The data were processed with Cell Quest Software (Becton Dickinson, USA).

#### 2.5.2. Multipotent Differentiation Capabilities

iPSC-MSCs at a density of 2 × 10^4^ per well were seeded in 24-well plates in MSC culture medium (DMEM/F12 + 10% FBS + 2 mM L-glutamine). After 2 days of culture, the MSC medium was replaced with osteogenic medium, adipogenic medium, and chondrogenic medium, respectively. After 14 days of culture, the cells were fixed with 4% PFA. For osteogenic differentiation, cells were cultured in MSC culture medium supplemented with osteogenic supplement (Gibco, USA) for 14 days and detected by alkaline phosphatase (ALP) staining (MesGen Biotech, China). For adipogenic differentiation, cells were cultured in MSC culture medium supplemented with an adipogenic supplement (Gibco, USA) for 14 days and incubated with a 0.5% oil red O (ORO) solution (MesGen Biotech, China). For chondrogenic differentiation, cells were cultured in MSC culture medium supplemented with chondrogenic supplement (Gibco, USA) for 14 days. After differentiating in monolayer, cells were incubated with a 0.1% toluidine blue (TB) solution (MesGen Biotech, China). Nondifferentiated iPSC-MSCs were used as a control.

#### 2.5.3. Gene Expression Analyses

Quantitative real-time PCR (qRT-PCR) was carried out as described to detect the pluripotent activity of iPSCs at P14 and iPSC-MSCs at P7 [18]. Specific pluripotent genes NANOG, OCT-4, and MSX-1 were assayed. Total RNA was extracted using TRIzol (Sangon, Shanghai), and cDNA was synthesized by the PrimeScript® RT reagent kit (Thermo, USA). qRT-PCR was performed using SYBR Green (Takara, Japan) and analyzed by a 7500 fast real-time PCR system (LIFE, USA). Glyceraldehyde-3-phosphate dehydrogenase (GAPDH) was used to standardize the relative expression of target genes. The reaction conditions were as follows: predenaturation at 95°C for 15 min, denaturation at 95°C for 15 s, annealing at 56°C for 32 s, and extension at 72°C for 32 s, for 40 cycles. The relative expression of each target gene was calculated according to 2^−ΔΔct^ [24]. The primers are designed and synthesized by the Sangon Biotech Co., Ltd (Shanghai, China). The sequences of the primers used are listed in Table 1.

#### 2.5.4. Evaluation of Cell Self-Renewal Ability

Cell proliferation was evaluated by the CCK-8 assay. The iPSC-MSCs at different passages (P1, P3, P5, P7, and P9) were seeded in 96-well plates cultured with MSC culture medium. At 1, 2, 3, and 4 days, the CCK-8 working solution was added according to the manufacturer's procedure. The OD values were recorded at 450 nm by a plate reader.

#### 2.5.5. Teratoma Formation Test

To test whether the derived iPSC-MSCs are relatively safe, we conducted an *in vivo* assay. Previous research had shown that undifferentiated iPSCs produced teratomas *in vivo* [18], so only the teratoma formation of iPSC-MSCs was assayed. 100 *μ*L iPSC-MSCs with a density of 1 × 10^7^ cells/mL were injected subcutaneously into the hind limbs of four-week-old male BALB/C nude mice (*n* = 3), and mice in the control group were injected with the same volume of saline. Teratoma formation was examined by visual inspection and photography after transplantation for 4 weeks. [25] One injection per mice was performed.

### 2.6. Osteogenesis of iPSC-MSCs

iPSC-MSCs suspension at P7 was added to the well with the O-ECM or O-ECM/PLLA/SF nanofibrous scaffolds at a density of 2 × 10^4^ cells/mL in a culture medium (DMEM/F12 + 10% FBS + 1% penicillin/streptomycin + 2 mM L-glutamine). As a positive control, the same iPSC-MSCs was added to the well with the O-ECM/PLLA/SF nanofibrous scaffolds and osteogenic medium (OM) containing 10 mM *β*-glycerophosphate (Sigma), 10 nM dexamethasone (Sigma), and 50 *μ*g/mL ascorbic acid (Sigma). The same iPSC-MSCs were seeded on the TCP with a culture medium (DMEM/F12 + 10% FBS + 1% penicillin/streptomycin + 2 mM L-glutamine). And the culture media were changed every 2 days [22].

#### 2.6.1. Expression of Osteogenic Genes

Osteogenic genes OCN, Runx2, and Col-1 expressed by iPSC-MSCs cultured on the nanofibrous scaffolds for 2 weeks were evaluated. The sequences of the primers used are listed in Table 1. qRT-PCR was performed following the same procedure as described above.

#### 2.6.2. Immunofluorescence Staining

The specimens (*n* = 5) were fixed with 4% PFA for 30 min and permeated in 0.5% Triton X-100 for 20 min. Subsequently, the samples were blocked in 2% BSA (MesGen Biotech, China) for 30 min. Then, the samples were incubated with rabbit monoclonal antibodies against OPN and Col-1 (all from Abcam, USA) at a dilution of 1 : 200 overnight at 4°C, followed by incubation with goat anti-rabbit polyclonal antibody Alexa Fluor 488 (Invitrogen, USA) at a dilution of 1 : 200 for 2 h in the dark. Finally, cell nuclei were stained by DAPI (MesGen Biotech, China) for viewing under a fluorescence microscope (LSM 710, JEOL, Japan).

#### 2.6.3. Alizarin Red S (ARS) Staining

The samples (*n* = 5) were fixed with 4% PFA for 10 min at room temperature. PBS was used to wash the plate several times, and cells were incubated with 2% ARS (MesGen Biotech, China) for 30 min at dark [26]. The images were obtained using a fluorescence microscope.

#### 2.6.4. Collagen Quantitative Analysis

The samples (*n* = 5) were lysed in 1% Triton X-100 for 15 min, and the lysate was collected. Collagen content was determined by a hydroxyproline kit (Nanjing Jiancheng Bioengineering Institute, Nanjing, China) following the manufacturer's instructions. The OD values were recorded at 550 nm by a plate reader (Multiskan MK3, Thermo, USA).

### 2.7. Bone Regeneration, *In Vivo*

#### 2.7.1. Seeding iPSC-MSCs on O-ECM/PLLA/SF Scaffolds

iPSC-MSCs were collected by 0.25% trypsin-EDTA, and single cell suspensions of P7 at a density of 1 × 10^6^ cells/mL were seeded on the O-ECM/PLLA/SF nanofibrous scaffolds. After 2 days of culture, O-ECM/PLLA/SF carried with iPSC-MSCs was used for animal experiment.

#### 2.7.2. Construction of a Cranial Bone Defect Model

A rat cranial bone defect model was designed by the method described previously with minor modifications [27]. In brief, thirty 6-week-old male SD rats (*n* = 5) were intraperitoneally injected with pentobarbital (50 mg/kg). A 5 mm diameter defect was created on the skull bones. Sterile nanofibrous scaffolds (PLLA/SF, O-ECM/PLLA/SF, and O-ECM/PLLA/SF carried with iPSC-MSCs) were transplanted into the defects, respectively. After operation, adequate food and water were supplied, and the wound was treated with iodophors every day. The SD rats were killed by anesthetic, and the defects were examined at 4 and 8 weeks after implantation.

#### 2.7.3. Microcompute Tomography

Dual-source CT (SOMATOM Definition, USA) was used to detect the bone regeneration of the defects. Under general anesthesia, the rats were fixed and scanned over their entire length at a resolution of 20 *μ*m.

#### 2.7.4. Histological Analysis and Immunohistochemical Staining

The samples (*n* = 3) were fixed with 4% PFA for 24 h and decalcified in a 12% EDTA solution for 2 weeks. Next, the samples were washed, dehydrated by a series of different concentrations of ethanol, and finally embedded in paraffin to make sections. After dewaxing, the sections were dyed with hematoxylin and eosin (H&E) reagent, Masson's trichrome reagent, and an immunohistochemical reagent to determine ECM, collagen, and protein expression, respectively. The images were observed under a microscope.

### 2.8. Statistical Analysis

All experimental data were presented as the means ± standard deviations and analyzed through one-way analysis of variance (ANOVA) followed by Tukey's test. The normal distribution was confirmed with a Shapiro–Wilk normalization test. Statistical analysis was processed using the Origin 8.0 statistical software. The statistical significance was considered when ^∗^*p* < 0.05 and ^∗∗^*p* < 0.01.

## 3. Results

### 3.1. Fabrication and Characterization of PLLA/SF Nanofibrous Scaffolds

**Figure 2:**
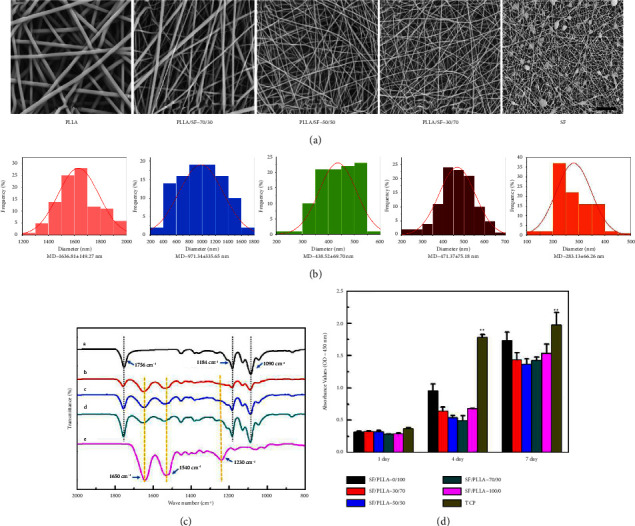
(a) SEM images of PLLA/SF nanofibrous scaffolds with the different mass ratios including 100/0, 70/30, 50/50, 300/70, and 0/100. (b) The diameter distributions of PLLA/SF nanofibrous scaffolds with the different mass ratios were investigated. MD, mean diameter. (c) Fourier transmission infrared spectrum (FTIR) of different mass ratios of PLLA/SF nanofibrous scaffolds. A, pure PLLA; B, PLLA/SF = 70/30; C, PLLA/SF = 50/50; D, PLLA/SF = 30/70; E, pure SF. (d) The biocompatibility of PLLA/SF nanofibers was measured as the OD values using L929 cells and cell counting kit-8. TCP, tissue culture plates. Scale bar = 10 *μ*m. ^∗∗^*p* < 0.01.

As can be seen from Figure 2(a), all scaffolds were constituted of randomly distributed nanofibers and thoroughly interconnected porous structures. Bead-like defects were observed in the pure SF group.

The average fiber diameter of PLLA/SF nanofibrous scaffolds ranged from 1790 nm to 350 nm with increasing SF ratio (Figure 2(b)). The average diameter of the different PLLA/SF nanofibrous scaffolds was 1638.81 ± 149.27 nm (PLLA/SF = 100/0), 971.34 ± 333.65 nm (PLLA/SF = 70/30), 438.52 ± 69.70 nm (PLLA/SF = 50/50), 471.37 ± 75.18 nm (PLLA/SF = 30/70), and 283.13 ± 66.26 nm (PLLA/SF = 0/100), respectively. The fiber diameter tended to be more homogeneous when increasing SF (ratio 70/30 compared to 30/70).

FTIR results showed the characteristic structures of PLLA/SF nanofibrous scaffolds with different mass ratios (Figure 2(c)). Among them, a–e correspond to the trend of absorption peaking from a pure PLLA group to the pure SF group, respectively. The peak at 1756 cm^−1^ corresponded to the stretching vibration of C=O, and the peaks at 1184 cm^−1^ and 1090 cm^−1^ were assigned to the asymmetric stretching of C-O and stretching vibration of C-O-C, respectively. The characteristic absorption peaks of SF at 1627 cm^−1^, 1517 cm^−1^, and 1230 cm^−1^ correspond to the amide I region of SF, the *α*-helix structure of the amide II region, and the amide III region, respectively.

To investigate the biocompatibility of the PLLA/SF nanofibrous scaffolds, the cell viability was examined with the CCK-8 assay over 1, 4, and 7 days. L929 cells showed proliferation in all groups, and no significant difference in cell viability was observed on different PLLA/SF nanofibrous scaffolds, indicating that all of the nanofibrous scaffolds were nontoxic and biocompatible for the survival of L929 cells. In addition, the proliferation of L929 cells in TCP was clearly higher and statistically significant from the other conditions at days 4 and 7 (Figure 2(d)).

Based on the above experimental data, PLLA/SF (50/50) nanofibrous scaffolds were selected for further study.

### 3.2. Osteoblast Culture and Build O-ECM/PLLA/SF Composite Scaffolds

**Figure 3:**
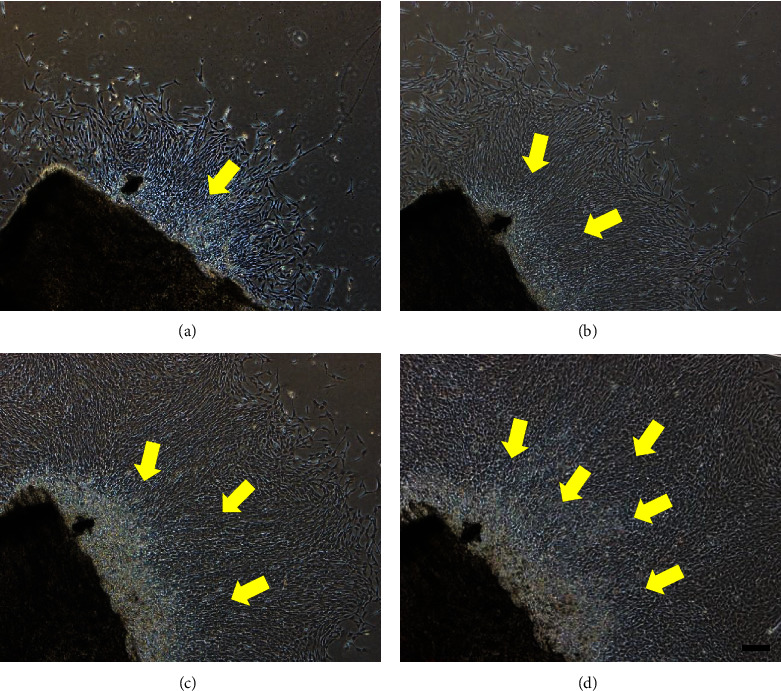
The morphology of osteoblasts cultured for 2 days (a), 3 days (b), 4 days (c), and 5 days (d). Scale bar = 200 *μ*m.

The 1-day-old mouse skulls were cultured on tissue culture plates. As shown in Figures 3(a)–3(d), plenty of active osteoblast-like cells crawled out of the bone over 2–5 days, and the cells formed the typical fibrous and/or spindle-shaped preliminary, exhibiting the typical osteoblasts' morphology [28]. After 10 days culture on PLLA/SF scaffolds, the process to fabricate O-ECM-coated PLLA/SF scaffolds was performed as in our previous study, which showed the acellular matrix was suitable for adhesion and osteogenic differentiation of MSCs [22].

### 3.3. Induction and Evaluation of iPSC-MSCs

**Figure 4:**
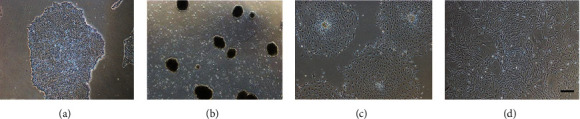
(a) The morphology of undifferentiated human iPSCs. (b) Embryoid bodies were found in suspension culture. (c) Fibroblast-like cells developed from the adhered EBs. (d) The morphology of purified iPSC-MSCs. Scale bar = 200 *μ*m.

After 5 days of culture, human iPSCs showed a smooth surface with well-defined borders, demonstrating an undifferentiated state (Figure 4(a)). Then, human iPSCs were cultured as floating EBs in the presence of 5 mM all trans retinoic acid for 2 days, and EBs were found in suspension culture (Figure 4(b)). Subsequently, the suspended EBs were plated onto 0.1% gelatin-coated 6-well cell culture plates to develop fibroblast-like cells (Figure 4(c)). Finally, the new cells from EBs were collected and cultured for up to 7 days and reached confluence (Figure 4(d)).

**Figure 5:**
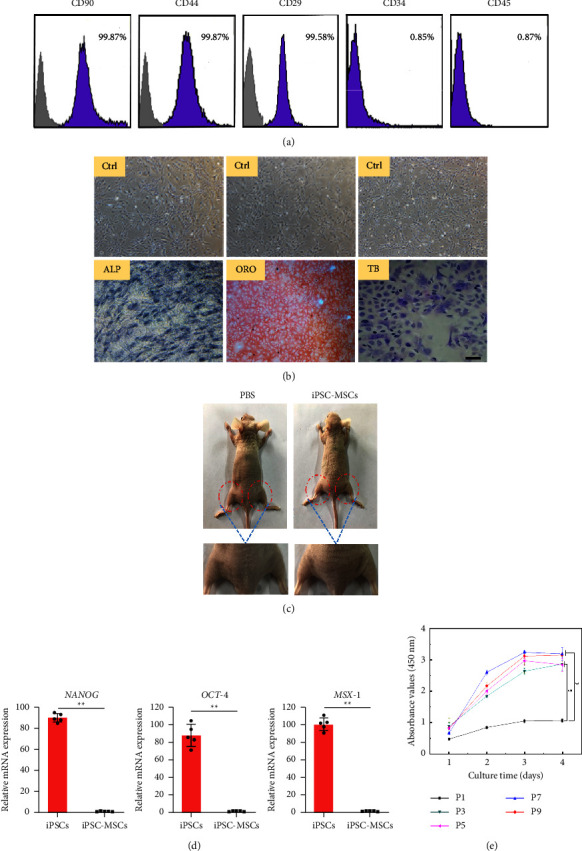
(a) Flow cytometry analysis of the expression of cell surface markers. (b) Alkaline phosphatase (ALP) staining of nondifferentiated iPSC-MSCs and differentiated iPSC-MSCs; oil red o (ORO) staining of nondifferentiated iPSC-MSCs and differentiated iPSC-MSCs; toluidine blue (TB) staining of nondifferentiated iPSC-MSCs and differentiated iPSC-MSCs. (c) No teratoma formation on the hind limbs of nude mice transplanted with PBS and iPSC-MSCs for 4 weeks. (d) Analysis of pluripotent gene expression in iPSC-MSCs. (e) Proliferation of iPSC-MSCs at different passages (P1, P3, P5, P7, and P9). Scale bar = 100 *μ*m. ^∗∗^*p* < 0.01.

As shown in Figure 5(a), the human iPSC-derived cells were positive for CD90 (99.87%), CD44 (99.87%), and CD29 (99.58%) and negative for CD34 (0.85%) and CD45 (0.87%). Based on these data, we define the cells derived from human iPSCs as MSCs [29]. ALP, oil red O, and toluidine blue staining demonstrated that the cells were able to differentiate into osteogenic, adipogenic, and chondrogenic lineages after 14 days of induction (Figure 5(b)). As shown in Figure 5(c), visual inspection alone was used to examine teratoma formation and no teratoma formation was found both in the iPSC-MSCs group and PBS group after injection with iPSC-MSCs or PBS for 4 weeks. qRT-PCR analysis showed that the expressions of NANOG, OCT-4, and MSX-1 were reduced significantly in the iPSC-MSCs group compared to the iPSCs group (Figure 5(d)). Finally, we cultured iPSC-MSCs at different passages (P1, P3, P5, P7, and P9) in 96-well plates and examined cell viability by CCK-8. As shown in Figure 5(e), cell proliferation curve revealed that the iPSC-MSCs at passage 1 proliferated slowly. The proliferation of iPSC-MSCs at P3, P5, P7, and P9 was clearly higher and statistically significant than the iPSC-MSCs at P1. The proliferation of iPSC-MSCs at P3 and P5 had no significant difference from the iPSC-MSCs at P7 and P9. The cell proliferation ability was enhancing gradually as the cell passage increased.

### 3.4. Osteogenic Differentiation, *In Vitro*

**Figure 6:**
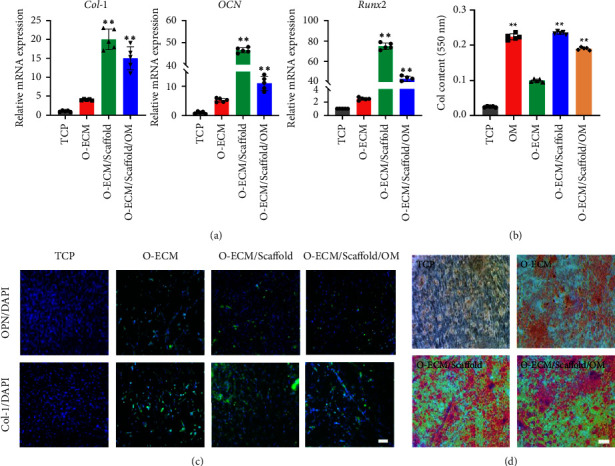
(a) Analysis of gene expression of Col-1, OCN, and Runx2 on different scaffolds after 14 days of culture in iPSC-MSC medium and osteogenic medium, respectively. TCP, tissue culture plates; O-ECM, osteoblast cell-derived extracellular matrix; Scaffold, PLLA/SF (50/50) nanofibers; OM, osteogenic medium. (b) Quantification of collagen by iPSC-MSCs cultured on different nanofibers after 14 days. (c) Immunofluorescence staining of OPN and Col-1 on different scaffolds after 14 days of culture in iPSC-MSC medium and osteogenic medium, respectively. (d) Alizarin red s (ARS) staining mages of iPSC-MSCs cultured on different scaffolds for 14 days. Scale bar = 100 *μ*m. ^∗∗^*p* < 0.01.

Our previous studies have shown that O-ECM was a grid structure with many protein deposits and coarse collagen components on scaffolds, and the presence of O-ECM on PLLA/SF scaffolds showed similar matrix moiety compared with the O-ECM on TCP [22]. Osteogenic relative gene expression levels in the O-ECM/PLLA/SF group were higher than the other groups (Figure 6(a)). The subsequent quantitative test of collagen presented a similar trend (Figure 6(b)). The expression level of the collagen in the O-ECM/PLLA/SF group was also the most prolific compared with the other groups. As shown in Figure 6(c), OPN and Col-1 produced a strong fluorescence intensity in the O-ECM/PLLA/SF group. In addition, OPN and Col-1 expressed a dot-inlay morphology on O-ECM/PLLA/SF nanofibrous scaffolds. The ARS results confirmed that the cells showed more intensity of red color when the cells were cultured on two scaffold groups, O-ECM/PLLA/SF and O-ECM/PLLA/SF supplemented with OM (Figure 6(d)). All these results indicated that an O-ECM-coated PLLA/SF nanofibrous scaffold could promote the osteogenic differentiation of cultured iPSC-MSCs.

### 3.5. Bone Regeneration, *In Vivo*

**Figure 7:**
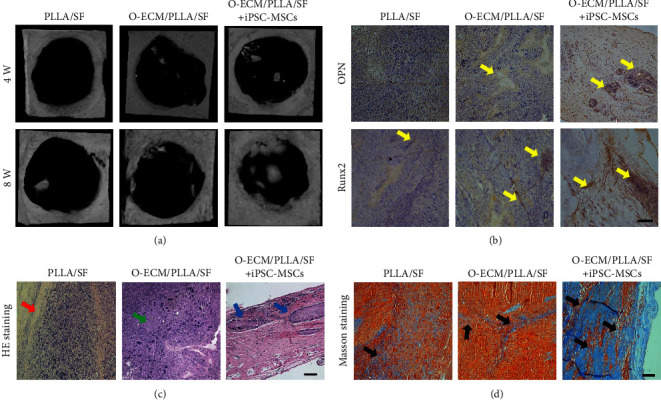
(a) Micro-CT images of rat cranial bone defects repaired by PLLA/SF, O-ECM/PLLA/SF, and O-ECM/PLLA/SF carried with iPSC-MSCs scaffolds 4 and 8 weeks after surgery. (b) The expressions of OPN and Runx2 via immunohistochemical staining. Yellow arrows indicate newly formed bone. (c) H&E staining images of different sample groups (8 weeks after surgery). Red arrow indicates implanted scaffolds, green arrow indicates scaffold fiber, and blue arrows indicate newly formed bone. (d) Masson's trichrome staining images of different sample groups (8 weeks after surgery). Black arrows indicate newly formed bone. Scale bar = 100 *μ*m.

The new bone generation at the fourth and eighth weeks after surgery was detected by micro-CT. As shown in Figure 7(a), the presented white areas directly represent the newly formed bone. From the subjective assessment of the results, at the fourth week, there was no newly formed bone in the PLLA/SF group. Whereas, in two O-ECM-coated scaffold groups, the newly formed bone existed at the edges of the defects. At the eighth week, there was a newly formed bone in the PLLA/SF group. The white areas in the two O-ECM-coated scaffold groups were slightly larger. However, the newly formed bone in the O-ECM/PLLA/SF carried with the iPSC-MSCs group not only had a thicker edge but also simultaneously existed in the center of the defect. As shown in Figure 7(b), the OPN and Runx2 (brown) expressed in the O-ECM/PLLA/SF carried with the iPSC-MSCs group were higher than those in the O-ECM/PLLA/SF group, which were significantly superior to the PLLA/SF group. In the PLLA/SF group, only a weak expression of Runx2 was observed. H&E (Figure 7(c)) and Masson's trichrome staining (Figure 7(d)) both showed that in the O-ECM/PLLA/SF carried with iPSC-MSCs group, a lot of newly formed bone was clearly observable in the defect sites, but only little newly formed bone was observed in the O-ECM/PLLA/SF group and fibrous tissue was observed in the PLLA/SF group. In addition, results from the histological staining showed that no teratoma formation was found in the O-ECM/PLLA/SF carried by the iPSC-MSCs group.

## 4. Discussion

At present, fabricated biomimetic scaffolds for bone tissue engineering typically suffer from poor osseointegration and osteogenesis, driving the need for developing new scaffolds to enhance mechanical properties, promote osteogenic differentiation, and further yield a large amount of bone tissue [30]. Advances in electrospinning technology have allowed bone-engineered scaffolds to possess nano-micro topography to aid cellular attachment, growth, interaction, and migration [31]. The electrospun scaffold was reported to have a resemblance to the structure of the extracellular matrix and could be used as a promising scaffold for bone tissue engineering applications [32]. The aim of this study was to create clinically relevant grafts involving PLLA/SF composite scaffolds coated with osteoblast-derived extracellular matrix that can be used therapeutically.

PLLA, as a biocompatible material with good mechanical integrity, has been electrospun to be nano/microfibres fulfill the mechanical strength requirements of bone tissue engineering *in vitro* and *in vivo* [33]. Some studies suggested that the PLLA nanofiber scaffold promotes progenitor stem cells' differentiation into osteoblasts, proliferation, and mineralization processes [34, 35]. SF is a natural protein derived from domesticated *Bombyx mori* cocoons. Due to its good biocompatibility and easy fabrication protocols, SF has been considered as an ideal biomaterial for kinds of tissue regeneration [36]. In this study, to overcome the shortcomings of the poor biocompatibility of PLLA, we used natural SF to achieve composite scaffolds with good hydrophilicity and biological compatibility. Here, we found that PLLA/SF has the best diameter distribution at the ratios of 50/50 and 30/70, closing to the scale of natural ECM [37]. Meanwhile, the fiber diameter tended to be more homogeneous when increasing SF (ratio 70/30 compared to 30/70). This phenomenon might be explained by the viscosity of the electrospinning solution, which decreased with the addition of silk fibroin, resulting in a corresponding decrease in fiber diameter [38]. In the overall evaluation of surface morphology, diameter distribution, and cell compatibility, we found that the scaffolds performed best when the ratio of PLLA/SF is 50/50, which is the most suitable for application in bone tissue engineering.

ECM makes an important contribution for directing stem cell responses when it is immobilized on the surface of biomedical implants [39]. Our previous studies reported that the surface modification with cell-derived matrices showed great potential in reproducing the milieu of natural ECM [22]. Harvestine et al. also demonstrated that ECM-coated composite scaffolds promote the persistence and osteogenesis of MSCs [40]. It is noteworthy that such property of ECM could be quite important for osteogenesis requirements. Thus, constructing a proper extracellular microenvironment on the implant surface via surface modification to direct cellular behaviors, especially osteogenic differentiation, is crucial to bone-repairing materials [39]. In this study, neonatal mouse osteoblasts were cultivated on the prepared PLLA/SF scaffolds and then decellularized to obtain extracellular matrix, nominated as O-ECM. Then, the PLLA/SF scaffolds coated with O-ECM were applied to further simulate the microenvironment needed for osteogenesis.

Recently, the derivation of MSCs from iPSCs, nominated as iPSC-MSCs, may be one of the most widely available sources of stem cells and thus possess great potential in cell-based therapy and regenerative medicines [41]. Deriving MSCs from iPSCs before specific differentiation has the advantage of producing a source of multipotent progenitor cells. This strategy can potentially yield a great deal of progenitor cells to regenerate bone defects [18]. In the present study, iPSCs were first induced into EBs and MSCs. After expansion several through passages, iPSC-MSCs exhibited a uniform fibroblast-like morphology and expressed high levels of MSC surface markers, consistent with surface markers in previous studies on MSCs [42]. Differentiation experiments showed that iPSC-MSCs possessed the potential to differentiate into three different cell lineages: adipocytes, chondrocytes, and osteoblasts. These findings confirmed that the iPSC-MSCs generated in the present study are highly comparable and similar to MSCs. Meanwhile, no teratoma formation was found after subcutaneous injection of iPSC-MSCs 4 weeks *in vivo*, indicating that the biosafety of iPSC-MSCs had improved. Derivation and utilization of iPSC-MSCs in BTE may be a useful strategy to reduce or even eliminate the tumorigenicity after transplantation when compared with the direct use of iPSCs. Here, we found that O-ECM-coated PLLA/SF scaffolds could effectively promote the differentiation of human iPSC-MSCs into osteoblasts. Previous studies have also shown that electrospun nanofiber scaffolds coated with O-ECM significantly promote osteogenic differentiation of MSCs, but no further studies have been conducted *in vivo* [43]. Therefore, we cultured iPSC-MSCs on O-ECM-coated PLLA/SF scaffolds and then implanted the complexes into skull defected rat models to evaluate osteogenic differentiation and the capability of new bone formation. Although the appraisal of micro-CT results has been carried out through qualitative analysis, which makes it difficult to eliminate the influence of contrived factors, our data demonstrated that composite scaffolds coated with O-ECM supported cell adhesion, survival, and osteogenesis *in vivo*. The secretion of O-ECM by osteoblasts plays an important role in the construction and repair of bone tissue after injury. Such an approach could easily produce various tissue-specific ECM through different cell sheets and then immobilized them on various material surfaces through acellular techniques, which actually provides a novel strategy for biomedical surface modification on many biomaterials [39]. And these kinds of bionic scaffolds will hopefully be applied to other tissue engineering applications.

## 5. Conclusions

This study generated MSCs from human iPSCs and investigated the osteogenic differentiation and bone repair capabilities of iPSC-MSCs seeded on O-ECM-coated electrospun PLLA/SF scaffolds. iPSC-MSCs were generated by culturing iPSC colonies and EBs, which are highly similar to MSCs. PLLA/SF scaffolds coated with osteoblast cell-derived ECM enhanced the osteogenic differentiation potential of iPSC-MSCs, yielding highly elevated osteogenic marker expressions and bone mineral synthesis *in vitro*. The O-ECM/PLLA/SF scaffolds loaded with iPSC-MSCs were effective in promoting osteogenesis and repairing bone defects *in vivo*. These results demonstrate the promise of iPSC-MSCs for bone tissue engineering and the potential of iPSC-MSC-O-ECM/PLLA/SF construct to enhance bone regeneration. This is an initial approach to modifying the surface properties of electrospun nanofiber scaffolds for bone tissue engineering. Such a special O-ECM acquirement and immobilization method may establish a useful construct by being able to direct multiple stem cells for specific cell differentiation on various materials, showing promising potential in bone tissue engineering and other related biomedical applications.

## Figures and Tables

**Table 1:** Primers for qRT-PCR.

Genes	Primer sequence	Amplication size (bp)	Gene ID
NANOG	F: TGAACCTCAGCTACAAACAG	153	79923
	R: TGGTGGTAGGAAGAGTAAAG		

OCT-4	F: CCTCACTTCACTGCACTGTA	164	5460
	R: CAGGTTTTCTTTCCCTAGCT		

MSX-1	F: CGAGAGGACCCCGTGGATGCAGAG	307	4487
	R: GGCGGCCATCTTCAGCTTCTCCAG		

OCN	F: AGTCCAGCAAAGGTGCAGCC AGG	169	632
	R: TCAGCCAACTCGTCACAGTC		

Col-1	F: GCCAAGACGAAGACATC	138	1277
	R: AGATCACGTCATCGCACAAC CCT		

Runx2	F: CAGTAGATGGACCTCGGGAA	188	860
	R: AAATCACTGAGGCGGTC		

GAPDH	F: ATCCCATCACCATCTTCC	293	2597
	R: GAGTCCTTCCACGATACCA		

## Data Availability

The data used to support the findings of this study are available from the corresponding author upon request.
